# Genetic predisposition of six well‐defined polymorphisms in HMGB1/RAGE pathway to breast cancer in a large Han Chinese population

**DOI:** 10.1111/jcmm.12888

**Published:** 2016-05-31

**Authors:** Liling Yue, Qibing Zhang, Lan He, Minglong Zhang, Jing Dong, Dalong Zhao, Hongxing Ma, Hongming Pan, Lihong Zheng

**Affiliations:** ^1^ Department of BiogeneticsQiqihar Medical UniversityQiqiharHeilongjiangChina; ^2^ Department of General SurgeryDaqing Oilfield General HospitalDaqingHeilongjiangChina; ^3^ Department of MathematicsQiqihar Medical UniversityQiqiharHeilongjiangChina; ^4^ Clinical LaboratoryQiqihar Jianhua HospitalQiqiharHeilongjiangChina; ^5^ Clinical LaboratoryDaqing Oilfield General HospitalDaqingHeilongjiangChina; ^6^ Department of BiochemistryQiqihar Medical UniversityQiqiharHeilongjiangChina

**Keywords:** breast cancer, HMGB1/RAGE pathway, polymorphism, haplotype, genetic score

## Abstract

Breast cancer constitutes an enormous burden in China. A strong familial clustering of breast cancer suggests a genetic component in its carcinogenesis. To examine the genetic predisposition of high mobility group box‐1/receptor for advanced glycation end products (HMGB1/RAGE) pathway to breast cancer, we genotyped six well‐defined polymorphisms in this pathway among 524 breast cancer patients and 518 cancer‐free controls from Heilongjiang province, China. There were no deviations from Hardy–Weinberg equilibrium for all polymorphisms. In single‐locus analysis, the frequency of rs1800624 polymorphism mutant A allele in *RAGE* gene was significantly higher in patients than in controls (24.52% *versus* 19.50%, *P* = 0.006), with the carriers of rs1800624‐A allele being 1.51 times more likely to develop breast cancer relative to those with rs1800624‐GG genotype after adjustment (95% confidence interval or CI: 1.17–1.94, *P* = 0.001). In *HMGB1* gene, haplotype analysis did not reveal any significance, while in *RAGE* gene, haplotypes C‐T‐A and C‐A‐G (alleles in order of rs1800625, rs18006024, rs2070600) were significantly associated with an increased risk of breast cancer (adjusted OR = 2.72 and 10.35; 95% CI: 1.20–6.18 and 1.58–67.80; *P* = 0.017 and 0.015 respectively). In further genetic score analysis, per unit and quartile increments of unfavourable alleles were significantly associated with an increased risk of breast cancer after adjustment (odds ratio or OR = 1.20 and 1.26; 95% CI: 1.09–1.32 and 1.12–1.42; *P* < 0.001 and <0.001 respectively). Our findings altogether demonstrate a significant association between *RAGE* gene rs1800624 polymorphism and breast cancer risk, and more importantly a cumulative impact of multiple risk associated polymorphisms in HMGB1/RAGE pathway on breast carcinogenesis.

## Introduction

In recent two decades, breast cancer has escalated to an emerging epidemic and constitutes an enormous burden in China 1. Breast cancer now ranks as the sixth leading cause of cancer‐related mortalities in Chinese women, according to a latest report 2. A strong familial clustering of breast cancer suggests that a genetic component underpins its carcinogenesis 3, 4. Although great endeavours have been made to disentangle the genetic make‐up of breast cancer including genome‐wide association studies 5, 6, 7, the complete catalogue of driven genetic determinants is still unclear, which necessitates continuous exploration and perfection in subsequent bench practice. To enrich our knowledge in understanding the genetic basis of breast cancer, we in this study focused on the components of HMGB1/RAGE pathway to evaluate their genetic predisposition to the development of breast cancer in Chinese.

HMGB1 is the acronym for high mobility group box‐1, a proangiogenic nuclear cytokine implicated in tumorigenesis, proliferation and metastasis 8. A growing number of epidemiological studies have suggested that HMGB1 is linked to poor clinical pathologies in various human cancers 9, 10, 11. High mobility group box‐1 itself signals through the receptor for advanced glycation end products (RAGE) to trigger the activation of NFκB, the up‐regulation of leucocyte adhesion molecules and the production of proinflammatory cytokines and angiogenic factors 12. It is widely recognized that targeting HMGB1 and its receptor RAGE may represent a promising opportunity in cancer therapeutics 13, 14. There is compelling evidence from clinical and epidemiological studies suggesting that HMGB1 and RAGE are positive predictors for the onset and progression of breast cancer, as well as its metastasis and survival 9, 15, 16. However, it remains unclear whether the implication of HMGB1 and RAGE in breast carcinogenesis is genetically determined. To yield more information, we selected six well‐defined polymorphisms from the genes encoding *HMGB1* and *RAGE*, and examined their genetic predisposition to breast cancer in a large Han Chinese population from Heilongjiang province.

## Materials and methods

### Study population

This is a case–control association study. All study subjects were females of Han nationality, and they were enrolled from four local hospitals (Daqing Oilfield General Hospital, The 2nd and 3rd Affiliated Hospitals of Qiqihar Medical University and Qiqihar Jianhua Hospital) in Heilongjiang province, China between January 2013 and August 2015. This study was approved by the Ethics Committee of Qiqihar Medical University. All study subjects signed written informed consent before agreeing to participate in this study according to the Declaration of Helsinki.

### Diagnostic criteria

Breast cancer patients were newly diagnosed, histopathologically confirmed or previously untreated. There were no restrictions on age, gender and cancer‐stage at enrolment.

### Sample size

This study enrolled a total of 1042 female subjects, including 524 breast cancer patients and 518 cancer‐free controls.

### Data collection

For breast cancer patients, data were recorded on the age of first onset, age of menarche, menopausal age, family history of cancer, tumour size (T1‐T4), histological grade (G1‐G3) and lymph node. For controls, age at enrolment and age of menarche were recorded, and they reported to have no prior history of any cancer types (except for non‐melanoma skin cancer).

### Genomic DNA extraction

A blood heparinized sample was obtained from each study subject, and genomic DNA was extracted from leucocytes by the phenol‐cholesterol method according to a standard procedure.

### Polymorphism selection

Three polymorphisms (rs2249825, rs1412125, rs1045411) in *HMGB1* gene and three polymorphisms (rs1800625, rs18006024, rs2070600) in *RAGE* gene were selected for genotype determination and association analyses. These six polymorphisms were well defined and widely evaluated in association with a broad range of cancers 17, 18, 19, 20, 21.

### Genotype determination

The genomic sequences of six examined polymorphisms were amplified by polymerase chain reaction (PCR), and their genotypes were further distinguished by ligase detection reaction method 22. In detail, the primers for PCR were designed online at the website http://seq.yeastgenome.org/cgi-bin/web-primer. For each allele, a specific probe was synthesized, and an additional common probe capped with 6‐carboxy‐fluorescein at the 3′ end and with horylated at the 5′ end was also synthesized. The ligation reaction mixture contained PCR product (2 μl), 10× Taq DNA ligase buffer (1 μl), each discriminating probe (1 μM) and Taq DNA ligase (5 U) in double‐distilled water to make a volume of 10 μl. The ligation conditions were 30 cycles of 30 sec. at 94°C and of 3 min. at 56°C. After that, 1 μl ligation product was mixed with 1 μl of ROX passive reference and 1 μl of loading buffer before being denatured at 95°C for 3 min. and chilled rapidly in ice water. The fluorescent products of ligase detection reaction were differentiated using ABI 3730XL sequencer (Applied Biosystems, Foster city, CA, USA).

To test the validity and accuracy of this genotyping method, 48 DNA samples were randomly selected and genotyped for the second time by ligase detection reaction method, and reduplication results were 100% consistent. Genotyping was determined by laboratory workers in a manner blind to the case–control status and pertinent characteristics of study subjects.

### Statistical analysis

All examined polymorphisms were checked for adherence to Hardy–Weinberg equilibrium by the chi‐squared test to avoid population stratification or genotyping misclassification. The genotype/allele distributions between patients and controls were compared by the chi‐squared test or Fisher's exact test where appropriate. Besides overall comparisons, stratified analyses according to the median values of age and age of menarche among all subjects were also conducted for the genotype/allele distributions between the two groups. The risk prediction for breast cancer was quantified by Logistic regression analyses before and after controlling for confounding factors (age and age of menarche). Effect‐size estimates were expressed as odds ratio (OR) and its 95% confidence interval (95% CI).

Considering the fact that the impact of a single polymorphism might be small, the co‐occurrence of unfavourable alleles of multiple polymorphisms can enhance the risk for breast cancer. A genetic score is therefore created on the basis of the number of unfavourable alleles for each subject by assigning zero, one or two unfavourable alleles of each polymorphism and summing them up. Besides per score increment, total genetic score was also collapsed into quartiles, and risk prediction was quantified by Logistic regression analyses before and after controlling for confounding factors. In addition, haplotype analysis is proven to be more informative than studying the role of polymorphisms independently. Haplotype analysis was undertaken within each gene under a generalized linear model by using the HAPLO.STATS program before and after controlling for confounding factors. The HAPLO.STATS program was implemented in the R Project for Statistical Computing version 2.6.2 (available at the website www.r-project.org/).

Unless otherwise stated all statistical analyses were carried out by Stata software version 13.0 (StataCorp LP, College Station, TX, USA). The power to reject null association was calculated by the PS: Power and Sample Size Calculation software version 3.0 (Copyright © 1997‐2009 by William D. Dupont and Walton D. Plummer) 23.

## Results

### Baseline characteristics

There were 524 breast cancer patients and 518 controls in this study, and their baseline characteristics are listed and compared in Table 1. Controls tended to be older than patients (56.49 years *versus* 53.76 years, *P* < 0.001). In contrast, the mean age of menarche was higher in patients than in controls (14.61 years *versus* 13.04 years, *P* < 0.001). The mean menopausal age was 50.19 years in patients, and about 6% of patients had a positive family history of cancers. As for tumour size, there were 49.69%, 42.77%, 3.77% and 3.77% of patients having T1, T2, T3 and T4 respectively. With regard to tumour stage, the G2 (49.27%) and G3 (46.10%) stages accounted for the majority of breast cancer patients. A total of 42.47% of patients were detected with positive lymph node.

**Table 1:** The baseline characteristics of study subjects

Characteristics	Patients	Controls	*P*
Sample size	524	518	
Age (years)	53.76 (12.62)	56.49 (10.04)	<0.001
Age of menarche (years)	14.61 (1.65)	13.04 (1.12)	<0.001
Menopausal age (years)	50.19 (3.98)	n.a.	
Family history of cancer	5.95%	0.00%	<0.001
Tumour size			
T1	49.69%	n.a.	
T2	42.77%		
T3	3.77%		
T4	3.77%		
Tumour stage			
I	4.63%	n.a.	
II	49.27%		
III	46.10%		
Positive lymph node	42.47%	n.a.	

n.a.: not available. Data are expressed as mean (S.D.) or percentage.

### Single‐locus analysis

The chi‐squared‐based goodness‐of‐fit test revealed that the genotype distributions of six examined polymorphisms did not deviate from Hardy–Weinberg equilibrium at a significance level of 5%. As shown in Table 2, the genotype distributions of rs1800624 polymorphism in *RAGE* gene differed significantly between patients and controls (*P* = 0.008), and the frequency of its mutant A allele was significantly higher in patients than in controls (24.52% *versus* 19.50%, *P* = 0.006), even after the Bonferroni correction (*P* < 0.05/6). The power to reject the null hypothesis of no allelic difference for rs1800624 polymorphism between patients and controls was estimated to be 82.9%. In addition, for rs2249825 and rs1800625 polymorphisms, there was marginal significance in allele distributions between the two groups (*P* = 0.024 and 0.029, respectively), and after the Bonferroni correction, no significance was found.

**Table 2:** The genotype distributions and allele frequencies of six examined polymorphisms in HMGB1/RAGE pathway between breast cancer patients and controls

Polymorphisms	Class	WW	WM	MM	*P*_chi‐squared_	W (%)	M (%)	*P*_chi‐squared_
rs2249825		CC	CG	GG		C	G	
	Patients	462	61	1	0.069	93.99	6.01	0.024
	Controls	432	83	3		91.41	8.59	
rs1412125		TT	TC	CC		T	C	
	Patients	281	213	30	0.363	73.95	26.05	0.170
	Controls	300	193	25		76.54	23.46	
rs1045411		GG	GA	AA		G	A	
	Patients	373	138	13	0.100	84.35	15.65	0.077
	Controls	389	124	5		87.07	12.93	
rs1800625		TT	TC	CC		T	C	
	Patients	330	174	20	0.081	79.58	20.42	0.029
	Controls	360	143	15		83.30	16.70	
rs1800624		TT	TA	AA		T	A	
	Patients	296	199	29	0.008	75.48	24.52	0.006
	Controls	341	152	25		80.50	19.50	
rs2070600		GG	GA	AA		G	A	
	Patients	310	158	56	0.052	74.24	25.76	0.616
	Controls	298	183	37		75.19	24.81	

WW: homozygous wild genotype; WM: heterozygous genotype; MM: homozygous mutant genotype; W: wild allele; M: mutant allele.

In further stratified analyses by age at a cut‐off value of 55 years (median), the allelic association with breast cancer risk was strikingly significant for *RAGE* gene rs1800624 polymorphism among subjects aged <55 years (*P* = 0.005) and for *HMGB1* gene rs2249825 polymorphism among subjects aged ≥55 years (*P* = 0.007; Table S1). Grouping subjects by age of menarche at a cut‐off value of 14 years (median) revealed only significant allelic association for *RAGE* gene rs1800624 polymorphism among subjects with age of menarche ≥14 years (*P* = 0.003; Table S2).

Given the small number of mutant homozygotes, only additive and dominant models were conducted for six examined polymorphisms (Table 3). The significant association was still noted for rs1800624 polymorphism, even after adjusting for age and age of menarche. For example, the carriers of rs1800624‐A allele were 1.51 times more likely to develop breast cancer relative to those with rs1800624‐GG genotype after adjusting for age and age of menarche (95% CI: 1.17–1.94, *P* = 0.001), even after the Bonferroni correction (*P* < 0.05/6). In addition for rs1800625 polymorphism, the mutant genotype conferred a marginally increased risk for breast cancer before and after adjusting for age and age of menarche, especially under the dominant model, that is, the odds of having breast cancer was 1.34 (95% CI: 1.04–1.73; *P* = 0.026) and 1.31 (95% CI: 1.01–1.69; *P* = 0.044) before and after adjustment. In contrast to rs2249825 polymorphism, the carriers of mutant genotype or allele had a reduced risk for breast cancer with marginal significance, which did not survive the Bonferroni correction.

**Table 3:** Risk prediction of single examined polymorphisms in HMGB1/RAGE pathway for breast cancer under additive and dominant models

Polymorphisms	OR; 95% CI; *P*	adj‐OR; 95% CI; *P* a
Additive model		
rs2249825	0.67; 0.48–0.95; 0.023	0.69; 0.49–0.97; 0.031
rs1412125	1.16; 0.94–1.42; 0.161	1.15; 0.94–1.42; 0.173
rs1045411	1.26; 0.98–1.61; 0.073	1.22; 0.95–1.58; 0.117
rs1800625	1.28; 1.03–1.60; 0.029	1.26; 1.00–1.58; 0.045
rs1800624	1.34; 1.09–1.64; 0.006	1.35; 1.09–1.66; 0.005
rs2070600	1.05; 0.87–1.26; 0.637	1.02; 0.84–1.23; 0.860
Dominant model		
rs2249825	0.67; 0.47–0.96; 0.028	0.69; 0.48–0.98; 0.039
rs1412125	1.19; 0.93–1.52; 0.164	1.19; 0.93–1.52; 0.169
rs1045411	1.22; 0.93–1.61; 0.154	1.18; 0.90–1.56; 0.232
rs1800625	1.34; 1.04–1.73; 0.026	1.31; 1.01–1.69; 0.044
rs1800624	1.48; 1.16–1.91; 0.002	1.51; 1.17–1.94; 0.001
rs2070600	0.94; 0.73–1.20; 0.593	0.90; 0.70–1.16; 0.417

a *P* was adjusted for age and age of menarche in Logistic regression models. OR: odds ratio; 95% CI: 95% confidence interval.

### Haplotype analysis

In theory, a haplotype is defined as the combination of multiple alleles on 1 chromosome. Haplotype analysis refers to the simultaneous analysis of multiple polymorphisms. Considering that the genes encoding *HMGB1* (13q12) and *RAGE* (6p21.3) are located on different chromosomes, haplotype analysis is conducted separately (Table 4). The most common haplotype was treated as the reference group in Logistic regression models. In *HMGB1* gene, haplotype analysis did not reveal any statistical significance, and haplotype C‐T‐A (alleles in order of rs2249825, rs1412125, rs1045411 polymorphisms), which was over‐represented in patients relative to controls (11.80% *versus* 8.96%, *P* = 0.045) and was marginally associated with breast cancer risk (adjusted OR = 1.37; 95% CI: 0.97–1.93, *P* = 0.074). In *RAGE* gene, when compared with the reference haplotype T‐T‐G (alleles in order of rs1800625, rs18006024, rs2070600 polymorphisms), two haplotypes, C‐T‐A and C‐A‐G, were over‐represented in patients, and were significantly associated with an increased risk of breast cancer even after adjusting for age and age of menarche (adjusted OR = 2.72 and 10.35; 95% CI: 1.20–6.18 and 1.58–67.80; *P* = 0.017 and 0.015).

**Table 4:** The frequencies of gene‐based haplotypes and their risk prediction for breast cancer

Haplotype	Patients	Controls	*P*	OR; 95% CI; *P*	adj‐OR; 95% CI; *P* a
*HMGB1* gene: rs2249825‐rs1412125‐rs1045411					
C‐T‐G	56.35%	58.99%	0.215	Reference group	Reference group
C‐C‐G	22.54%	21.20%	0.320	1.13; 0.89–1.43; 0.318	1.15; 0.90–1.45; 0.262
C‐T‐A	11.80%	8.96%	0.045	1.37; 0.97–1.92; 0.074	1.37; 0.97–1.93; 0.074
G‐T‐G	5.46%	6.88%	0.050	0.83; 0.56–1.23; 0.363	0.85; 0.57–1.25; 0.402
C‐C‐A	3.30%	2.26%	0.075	1.73; 0.82–3.65; 0.150	1.52; 0.71–3.25; 0.279
G‐T‐A	0.34%	1.72%	0.038	0.25; 0.05–1.29; 0.097	0.29; 0.06–1.28; 0.101
*RAGE* gene: rs1800625‐rs18006024‐rs2070600					
T‐T‐G	45.68%	45.75%	0.060	Reference group	Reference group
T‐T‐A	14.63%	19.26%	0.093	0.78; 0.58–1.05; 0.100	0.78; 0.58–1.05; 0.098
T‐A‐G	14.61%	15.31%	0.387	0.96; 0.71–1.29; 0.771	0.97; 0.72–1.31; 0.851
C‐T‐G	9.88%	13.73%	0.857	0.73; 0.51–1.04; 0.081	0.74; 0.52–1.06; 0.100
C‐T‐A	5.29%	1.76%	0.012	3.03; 1.31–7.00; 0.010	2.72; 1.20–6.18; 0.017
T‐A‐A	4.66%	2.98%	0.138	1.42; 0.89–2.25; 0.142	1.43; 0.90–2.28; 0.134
C‐A‐G	4.07%	0.41%	<0.001	1.27; 0.18–8.80; 0.014	10.35; 1.58–67.80; 0.015
C‐A‐A	1.18%	0.80%	0.053	1.43; 0.46–4.40; 0.537	1.30; 0.45–3.80; 0.626

a *P* was adjusted for age and age of menarche in the HAPLO.STATS program. OR: odds ratio; 95% CI: 95% confidence interval.

### Genetic score analysis

As some examined polymorphisms were significantly or marginally associated with breast cancer, a genetic score analysis was performed to evaluate the cumulative impact of risk associated polymorphisms, and the results are summarized in Table 5. In comparison with the first quartile as the reference group (fewer than four unfavourable alleles), the risk prediction for breast cancer increased exponentially with the increasing number of unfavourable alleles within the 2nd quartile (OR = 0.99; 95% CI: 0.74–1.34; *P* = 0.961), 3^rd^ quartile (OR = 1.35; 95% CI: 0.95–1.92; *P* = 0.090) and 4th quartile (OR = 2.21; 95% CI: 1.47–3.31; *P* < 0.001) after adjusting for age and age of menarche. In addition, per unit and quartile increments of unfavourable genotypes were significantly associated with an increased risk of breast cancer after adjustment (OR = 1.20 and 1.26; 95% CI: 1.09–1.32 and 1.12–1.42; *P* < 0.001 and <0.001 respectively).

**Table 5:** The distributions of unfavourable alleles in quartiles and their risk prediction for breast cancer

Number of unfavourable alleles	Patients	Controls	OR; 95% CI; *P*	adj‐OR; 95% CI; *P* a
1–3	194 (37.03%)	225 (43.43%)	Reference group	Reference group
4	137 (26.15%)	161 (31.08%)	0.99; 0.73–1.33; 0.931	0.99; 0.74–1.34; 0.961
5	101 (19.27%)	85 (16.41%)	1.38; 0.97–1.95; 0.070	1.35; 0.95–1.92; 0.090
6–10	92 (17.56%)	47 (9.08%)	2.27; 1.52–3.39; <0.001	2.21; 1.47–3.31; <0.001
Per unit increment			1.21; 1.11–1.33; <0.001	1.20; 1.09–1.32; <0.001
Per quartile increment			1.27; 1.13–1.43; <0.001	1.26; 1.12–1.42; <0.001

a *P* was adjusted for age and age of menarche in Logistic regression models. OR: odds ratio; 95% CI: 95% confidence interval.

## Discussion

In this case–control study, we examined the genetic predisposition of six well‐defined polymorphisms in HMGB1/RAGE pathway to breast cancer in a large Han Chinese population, and we observed a significant association between *RAGE* gene rs1800624 polymorphism and breast cancer. More importantly, further haplotype and genetic score analyses suggested that there was a cumulative impact of multiple risk associated polymorphisms in this pathway on the development of breast cancer.

Currently, evidence for the implication of activated HMGB1/RAGE pathway in cell proliferation, angiogenesis and metastasis during breast cancer progression is rapidly accumulating 24, 25, 26. This pathway has been proposed as a promising target for the prediction, prevention and treatment of breast cancer 16, 27, 28. It will be very intriguing to know how the components of HMGB1/RAGE pathway alter genetic susceptibility to breast cancer. There is a wide recognition that knowledge of an individual's genetic make‐up will facilitate personalized medicine, including risk stratification and further targeted preventative and therapeutic interventions 29. To fill this void in knowledge, we designed this study to test the hypothesis that the implication of HMGB1 and RAGE in breast carcinogenesis is genetically regulated by genotyping six well‐defined polymorphisms among 524 breast cancer patients and 518 cancer‐free controls from Heilongjiang province, China.

Candidate gene approach is a key research paradigm for unfolding the genetic underpinnings of complex diseases, including cancers 30. Adopting this approach, we observed that the mutant A allele of rs1800624 polymorphism in *RAGE* gene was significantly associated with an increased risk of having breast cancer in Han Chinese, in contrast to the negative findings for this polymorphism in a recent study by Pan *et al*. among 1013 local residents of Qiqihar city 17. However, a cautionary note should be sounded regarding the small difference of mutant allele frequency of rs1800624 polymorphism between breast cancer patients (24.52%) and controls (19.50%, *P* = 0.006) in this study, and the power to detect this significant association was around 80%. The association between rs1800624 polymorphism and breast cancer, albeit statistically significant, has to be interpreted with caution, and independent confirmation will be important. In addition, both this study and the study by Pan *et al*. 17 failed to identify the significant contribution of rs2070600 and rs1800625 polymorphisms to breast cancer risk. There is no doubt that risk assessment based on a single genetic locus is gravely insufficient, and the relative risk attributable to a single locus is usually small and hard to detect 31. To make up this shortcoming, we adopted haplotype and genetic score analyses to our data, and interestingly found a cumulative impact of multiple risk associated polymorphisms in this pathway on the development of breast cancer. Our findings therefore lend some credence to the claim that single gene or locus may not, by itself, exhibit a signification association with disease in all or most studies because its effect may be small and dependent on genotypes at other loci that can compensate for variation in the locus under study 32. As well exemplified by our haplotype analysis in *RAGE* gene, two haplotypes C‐T‐A and C‐A‐G (alleles in order of rs1800625, rs1800624 and rs2070600 polymorphisms), which differed only in the latter two loci, were observed to both confer an increased risk for breast cancer, a finding contradictory to the significant predominant role of rs1800624 polymorphism in single‐locus analysis. It is possible that the part played by rs2070600 polymorphism was not significant unless the coinheritance of the wild allele of rs1800624 polymorphism. To the best of our knowledge, this is the first study interrogating the combined association of *HMGB1* and *RAGE* genes with the risk of breast cancer, and further validation in other ethnic groups is required.

Finally, the interpretation and extrapolation of our findings must consider several potential limitations. The first limitation is the retrospective case–control association design, and such design cannot reveal the possible cause‐effect between HMGB1/RAGE pathway and breast cancer 33. The second limitation lays in the selection of only six polymorphisms from this pathway, and other variants especially low‐penetrance loci and copy number variations are of added interest. The third limitation is that our study subjects were enrolled from multiple hospitals, and population stratification might yield a selection bias. However, Hardy–Weinberg equilibrium test did not reveal any evidence of deviations for all examined polymorphisms, leaving the doubt of population stratification unlikely. The fourth limitation is that only genetic data of Han Chinese are analysed, and extrapolation of our findings to the other nationalities of China and other ethnic groups is speculative. For this reason, our findings need be validated in other populations.

Taken together, we through a genetic analysis of HMGB1/RAGE pathway observed a significant association between *RAGE* gene rs1800624 polymorphism and breast cancer risk, and more importantly, there was a cumulative impact of multiple risk associated polymorphisms in this pathway on the development of breast cancer.

## Funding

This study was financially supported by the Project of Department of Education of Heilongjiang Province (grant no. 12541903).

## Conflict of interest

None declared.

## Supporting information


**Table S1** The genotype distributions and allele frequencies of six examined polymorphisms in HMGB1/RAGE pathway between breast cancer patients and controls by age at a cut‐off value of 55 years (median).
**Table S2** The genotype distributions and allele frequencies of six examined polymorphisms in HMGB1/RAGE pathway between breast cancer patients and controls by age of menarche at a cut‐off value of 14 years (median).
