# Supplementary material for: Genetic predisposition of six well‐defined polymorphisms in HMGB1/RAGE pathway to breast cancer in a large Han Chinese population
Source: J Cell Mol Med. 2016 May 31;20(10):1966–73. doi: 10.1111/jcmm.12888 (PMC5020633; doi:10.1111/jcmm.12888)
Supplement: Supplementary file 1 — Table S1 The genotype distributions and allele frequencies of six examined polymorphisms in HMGB1/RAGE pathway between breast cancer patients and controls by age at a cut‐off value of 55 years (median). Table S2 The genotype distributions and allele frequencies of six examined polymorphisms in HMGB1/RAGE pathway between breast cancer patients and controls by age of menarche at a cut‐off value of 14 years (median). [file JCMM-20-1966-s001.doc]

**Supplementary Table S1.** The genotype distributions and allele frequencies of six examined polymorphisms in HMGB1/RAGE pathway between breast cancer patients and controls by age at a cutoff value of 55 years (median)

| **Polymorphisms** | **Class** | **WW** | **WM** | **MM** | **P_Chisq_** | **W (%)** | **M (%)** | **P_Chisq_** |
| --- | --- | --- | --- | --- | --- | --- | --- | --- |
| rs2249825 | Age≥55 years | CC | CG | GG | 0.013 | C | G | 0.007 |
|  | Patients | 207 | 22 | 0 |  | 95.20 | 4.80 |  |
|  | Controls | 238 | 49 | 2 |  | 90.83 | 9.17 |  |
|  | Age<55 years | CC | CG | GG | 0.809 | C | G | 0.575 |
|  | Patients | 255 | 39 | 1 |  | 93.05 | 6.95 |  |
|  | Controls | 194 | 34 | 1 |  | 92.14 | 7.86 |  |
| rs1412125 | Age≥55 years | TT | TC | CC | 0.083 | T | C | 0.030 |
|  | Patients | 119 | 96 | 14 |  | 72.93 | 27.07 |  |
|  | Controls | 177 | 101 | 11 |  | 78.72 | 21.28 |  |
|  | Age<55 years | TT | TC | CC | 0.928 | T | C | 0.728 |
|  | Patients | 162 | 117 | 16 |  | 74.75 | 25.25 |  |
|  | Controls | 123 | 92 | 14 |  | 73.80 | 26.20 |  |
| rs1045411 | Age≥55 years | GG | GA | AA | 0.160 | G | A | 0.283 |
|  | Patients | 169 | 55 | 5 |  | 85.81 | 14.19 |  |
|  | Controls | 221 | 67 | 1 |  | 88.06 | 11.94 |  |
|  | Age<55 years | GG | GA | AA | 0.515 | G | A | 0.253 |
|  | Patients | 204 | 83 | 8 |  | 83.22 | 16.78 |  |
|  | Controls | 168 | 57 | 4 |  | 85.81 | 14.19 |  |
| rs1800625 | Age≥55 years | TT | TC | CC | 0.602 | T | C | 0.481 |
|  | Patients | 151 | 69 | 9 |  | 81.00 | 19.00 |  |
|  | Controls | 196 | 86 | 7 |  | 82.70 | 17.30 |  |
|  | Age<55 years | TT | TC | CC | 0.028 | T | C | 0.022 |
|  | Patients | 179 | 105 | 11 |  | 78.47 | 21.53 |  |
|  | Controls | 164 | 57 | 8 |  | 84.06 | 15.94 |  |
| rs1800624 | Age≥55 years | TT | TA | AA | 0.341 | T | A | 0.201 |
|  | Patients | 129 | 88 | 12 |  | 75.55 | 24.45 |  |
|  | Controls | 181 | 94 | 14 |  | 78.89 | 21.11 |  |
|  | Age<55 years | TT | TA | AA | 0.007 | T | A | 0.005 |
|  | Patients | 167 | 111 | 17 |  | 75.42 | 24.58 |  |
|  | Controls | 160 | 58 | 11 |  | 82.53 | 17.47 |  |
| rs2070600 | Age≥55 years | GG | GA | AA | 0.573 | G | A | 0.747 |
|  | Patients | 143 | 65 | 21 |  | 76.64 | 23.36 |  |
|  | Controls | 172 | 94 | 23 |  | 75.78 | 24.22 |  |
|  | Age<55 years | GG | GA | AA | 0.037 | G | A | 0.450 |
|  | Patients | 167 | 93 | 35 |  | 72.37 | 27.63 |  |
|  | Controls | 126 | 89 | 14 |  | 74.45 | 25.55 |  |

Note: WW: homozygous wild genotype; WM: heterozygous genotype; MM: homozygous mutant genotype; W: wild allele; M: mutant allele.

**Supplementary Table S2.** The genotype distributions and allele frequencies of six examined polymorphisms in HMGB1/RAGE pathway between breast cancer patients and controls by age of menarche at a cutoff value of 14 years (median)

| **Polymorphisms** | **Class** | **WW** | **WM** | **MM** | **P_Chisq_** | **W (%)** | **M (%)** | **P_Chisq_** |
| --- | --- | --- | --- | --- | --- | --- | --- | --- |
| rs2249825 | M.age≥14 years | CC | CG | GG | 0.196 | C | G | 0.210 |
|  | Patients | 369 | 44 | 1 |  | 94.44 | 5.56 |  |
|  | Controls | 189 | 26 | 3 |  | 92.66 | 7.34 |  |
|  | M.age<14 years | CC | CG | GG | 0.470 | C | G | 0.432 |
|  | Patients | 93 | 17 | 0 |  | 92.27 | 7.73 |  |
|  | Controls | 243 | 57 | 0 |  | 90.50 | 9.50 |  |
| rs1412125 | M.age≥14 years | TT | TC | CC | 0.715 | T | C | 0.429 |
|  | Patients | 213 | 176 | 25 |  | 72.71 | 27.29 |  |
|  | Controls | 119 | 88 | 11 |  | 74.77 | 25.23 |  |
|  | M.age<14 years | TT | TC | CC | 0.963 | T | C | 0.806 |
|  | Patients | 68 | 37 | 5 |  | 78.64 | 21.36 |  |
|  | Controls | 181 | 105 | 14 |  | 77.83 | 22.17 |  |
| rs1045411 | M.age≥14 years | GG | GA | AA | 0.127 | G | A | 0.157 |
|  | Patients | 292 | 111 | 11 |  | 83.94 | 16.06 |  |
|  | Controls | 162 | 55 | 1 |  | 86.93 | 13.07 |  |
|  | M.age<14 years | GG | GA | AA | 0.799 | G | A | 0.637 |
|  | Patients | 81 | 27 | 2 |  | 85.91 | 14.09 |  |
|  | Controls | 227 | 69 | 4 |  | 87.17 | 12.83 |  |
| rs1800625 | M.age≥14 years | TT | TC | CC | 0.420 | T | C | 0.261 |
|  | Patients | 261 | 136 | 17 |  | 79.47 | 20.53 |  |
|  | Controls | 145 | 68 | 5 |  | 82.11 | 17.89 |  |
|  | M.age<14 years | TT | TC | CC | 0.150 | T | C | 0.159 |
|  | Patients | 69 | 38 | 3 |  | 80.00 | 20.00 |  |
|  | Controls | 215 | 75 | 10 |  | 84.17 | 15.83 |  |
| rs1800624 | M.age≥14 years | TT | TA | AA | 0.009 | T | A | 0.003 |
|  | Patients | 224 | 164 | 26 |  | 73.91 | 26.09 |  |
|  | Controls | 145 | 65 | 8 |  | 81.42 | 18.58 |  |
|  | M.age<14 years | TT | TA | AA | 0.456 | T | A | 0.626 |
|  | Patients | 72 | 35 | 3 |  | 81.36 | 18.64 |  |
|  | Controls | 196 | 87 | 17 |  | 79.83 | 20.17 |  |
| rs2070600 | M.age≥14 years | GG | GA | AA | 0.156 | G | A | 0.514 |
|  | Patients | 239 | 127 | 48 |  | 73.07 | 26.93 |  |
|  | Controls | 124 | 78 | 16 |  | 74.77 | 25.23 |  |
|  | M.age<14 years | GG | GA | AA | 0.425 | G | A | 0.349 |
|  | Patients | 71 | 31 | 8 |  | 78.64 | 21.36 |  |
|  | Controls | 174 | 105 | 21 |  | 75.50 | 24.50 |  |

Note: WW: homozygous wild genotype; WM: heterozygous genotype; MM: homozygous mutant genotype; W: wild allele; M: mutant allele; M.age: age of menarche.
